# Phosphodiesterase 10A is overexpressed in lung tumor cells and inhibitors selectively suppress growth by blocking β-catenin and MAPK signaling

**DOI:** 10.18632/oncotarget.20566

**Published:** 2017-08-27

**Authors:** Bing Zhu, Ashley Lindsey, Nan Li, Kevin Lee, Veronica Ramirez-Alcantara, Joshua C. Canzoneri, Alexandra Fajardo, Luciana Madeira da Silva, Meagan Thomas, John T. Piazza, Larry Yet, Brian T. Eberhardt, Evrim Gurpinar, Dennis Otali, William Grizzle, Jacob Valiyaveettil, Xi Chen, Adam B. Keeton, Gary A. Piazza

**Affiliations:** ^1^ Drug Discovery Research Center, Mitchell Cancer Institute, University of South Alabama, Mobile, Alabama, USA; ^2^ Department of Biochemistry and Molecular Biology, The University of Alabama at Birmingham, Birmingham, Alabama, USA; ^3^ Department of Chemistry, University of South Alabama, Mobile, Alabama, USA; ^4^ Department of Pharmacology, The University of Alabama at Birmingham, Birmingham, Alabama, USA; ^5^ Department of Pathology, The University of Alabama at Birmingham, Birmingham, Alabama, USA

**Keywords:** lung cancer, PDE10, PKG, cGMP, PQ10

## Abstract

Phosphodiesterase 10A (PDE10) is a cyclic nucleotide (e.g. cGMP) degrading enzyme highly expressed in the brain striatum where it plays an important role in dopaminergic neurotransmission, but has limited expression and no known physiological function outside the central nervous system. Here we report that PDE10 mRNA and protein levels are strongly elevated in human non-small cell lung cancer cells and lung tumors compared with normal human airway epithelial cells and lung tissue, respectively. Genetic silencing of PDE10 or inhibition by small molecules such as PQ10 was found to selectively inhibit the growth and colony formation of lung tumor cells. PQ10 treatment of lung tumor cells rapidly increased intracellular cGMP levels and activated cGMP-dependent protein kinase (PKG) at concentrations that inhibit lung tumor cell growth. PQ10 also increased the phosphorylation of β-catenin and reduced its levels, which paralleled the suppression of cyclin D1 and survivin but preceded the activation of PARP and caspase cleavage. PQ10 also suppressed RAS-activated RAF/MAPK signaling within the same concentration range and treatment period as required for cGMP elevation and PKG activation. These results show that PDE10 is overexpressed during lung cancer development and essential for lung tumor cell growth in which inhibitors can selectively induce apoptosis by increasing intracellular cGMP levels and activating PKG to suppress oncogenic β-catenin and MAPK signaling.

## INTRODUCTION

Lung cancer is the deadliest form of cancer for men and women of all races in the United States, causing more deaths than colorectal, breast, and prostate cancer combined [1, 2]. Lung cancer is usually aggressive and characterized by early progression and metastases, especially in patients diagnosed with non-small cell lung cancer (NSCLC). With chemotherapy providing only modest efficacy and associated with severe dose-limiting toxicities, new molecular targets are urgently needed to identify drug development candidates with potential for greater therapeutic efficacy and reduced toxicity [3-8].

Previous studies have shown that cyclic guanosine monophosphate (cGMP) signaling plays an important role in regulating tumor cell proliferation and survival [9-18]. Both particulate guanylyl cyclases (pGCs) and soluble guanylyl cyclases (sGCs) catalyze the biosynthesis of cGMP from GTP, while cGMP phosphodiesterases (PDEs) function to hydrolyze cGMP and terminate cGMP signaling [19, 20]. Activators of GCs or inhibitors of cGMP PDEs can increase intracellular cGMP levels to enhance the magnitude and/or duration of cGMP signaling to inhibit proliferation and induce apoptosis. Intracellular levels of cGMP are known to be aberrantly low in cancer cells resulting from the overexpression of one or more PDE isozymes, which may play an important role in tumorigenesis [9, 12, 17, 18]. The elevation of intracellular cGMP levels by cGMP PDE inhibitors can activate cGMP-dependent protein kinases (PKG), although the downstream pathways responsible for suppressing proliferation and inducing apoptosis of tumor cells are not well understood. Physiologically, cGMP signaling can be activated in the lung by natriuretic peptides binding to membrane receptors that can stimulate particulate GC activity, while nitric oxide activates soluble GC activity [19-22]. Although several cGMP degrading PDE isozymes have been reported in normal lung and lung tumors, including the cGMP-specific PDE isozyme, PDE5 [10, 18, 22, 23], the critical cGMP PDE isozymes that are induced during lung cancer progression and which are essential for tumor cell proliferation and survival have not been well studied, but may provide useful targets for the development of new anticancer drugs.

The human genome contains 21 PDE gene products grouped into 11 isozyme families having different substrate specificity, regulatory properties, tissue localization, and sensitivity to inhibitors [19, 20]. Compared with other PDE isozymes, the tissue distribution of PDE10 is limited with high expression in the brain striatum but low expression in most other normal tissues [20, 24-26]. Because PDE10 has been shown to play an important physiological role in the brain to affect motor function and cognition, PDE10 inhibitors have been developed and tested in clinical trials for the treatment of Huntington’s diseases and schizophrenia [27-29]. While there is no known physiological role of PDE10 in tissues outside the central nervous system, PDE10 may play an important role in lung disease as suggested by previous reports showing elevated PDE10 levels during pulmonary arterial hypertension [30].

We recently reported that PDE10 is elevated in colon tumor cell lines compared with primary cultures of cells derived from normal colonic mucosa [31]. PDE10 levels were also high in intestinal adenomas from ApcMin/+ mice, as well as human colon adenocarcinomas and metastatic lesions relative to normal intestinal mucosa [31, 32]. Consistent with the differential expression of PDE10 in normal and cancer cells, PDE10 inhibitors were found to selectively inhibit proliferation and induce apoptosis of colon tumor cells, while not affecting growth of normal colonocytes. PDE10 inhibition can increase intracellular levels of cGMP and cAMP to activate PKG and PKA signaling, respectively, although the mechanism responsible for its tumor cell growth inhibitory activity may be exclusively associated with cGMP/PKG signaling, given that cAMP PDE specific inhibitors (e.g. rolipram) or PKA activators (e.g. forskolin) do not inhibit colon tumor cell growth [12, 31-33]. Moreover, other studies reported that PKG inhibitors, but not PKA inhibitors, can attenuate the growth inhibitory activity of PDE10 inhibitors [32].

Here we show that PDE10 is overexpressed in NSCLC cell lines and human lung tumors compared with normal lung epithelial cells and tissues, respectively. Genetic silencing of PDE10 or inhibition by small molecules such as PQ10 selectively inhibits lung tumor cell growth and colony formation by activating cGMP/PKG signaling. We also show that PQ10 can phosphorylate β-catenin to induce degradation, leading to reduced levels of cyclin D1 and survivin and the induction of enzymes required for apoptosis. PQ10 can also suppress RAS-activated RAF/MAPK signaling within the same concentration range and kinetics as required to activate cGMP/PKG signaling.

## RESULTS

### PDE10 expression in human lung tumor and normal cells

**Figure 1:**
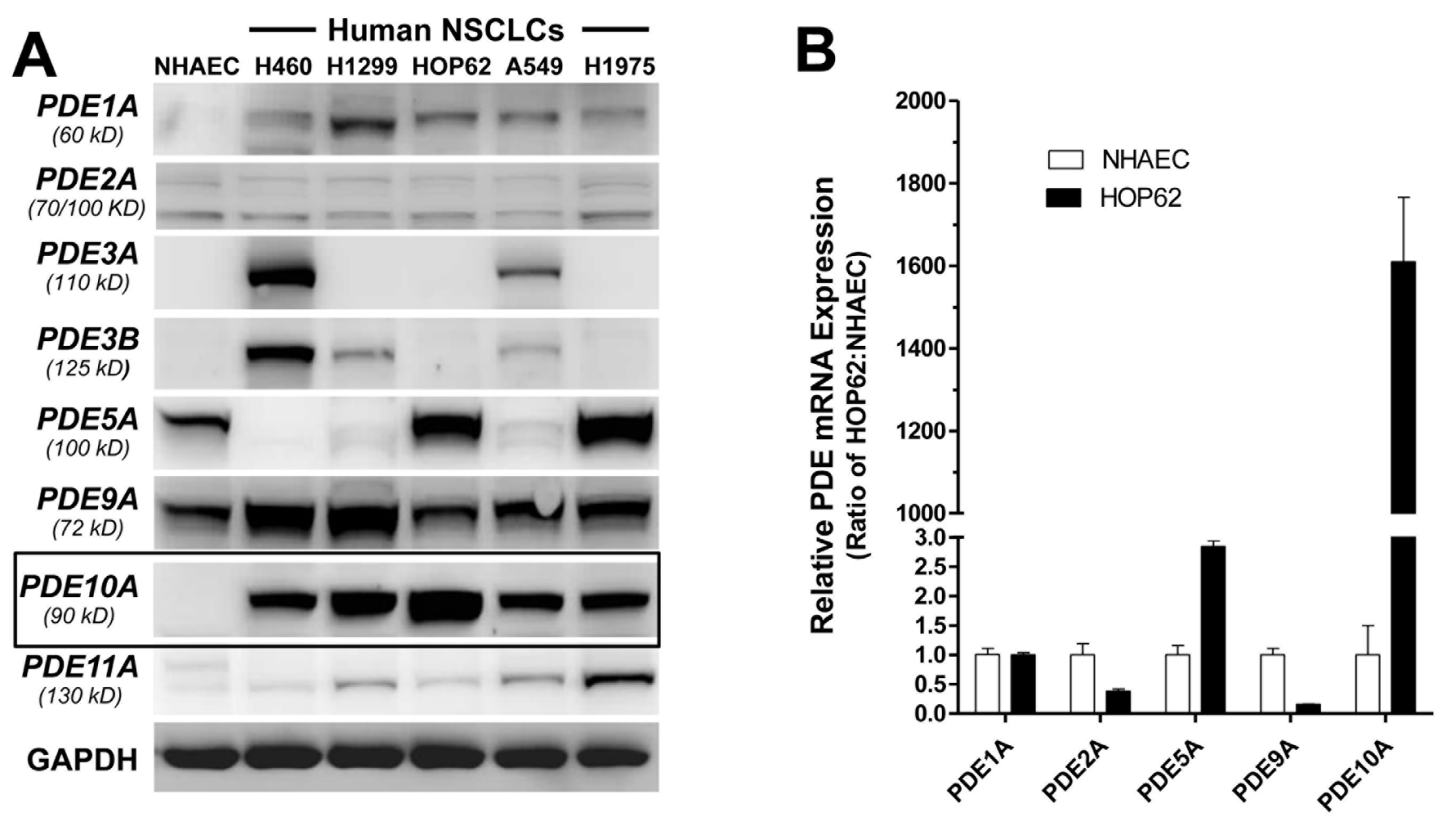

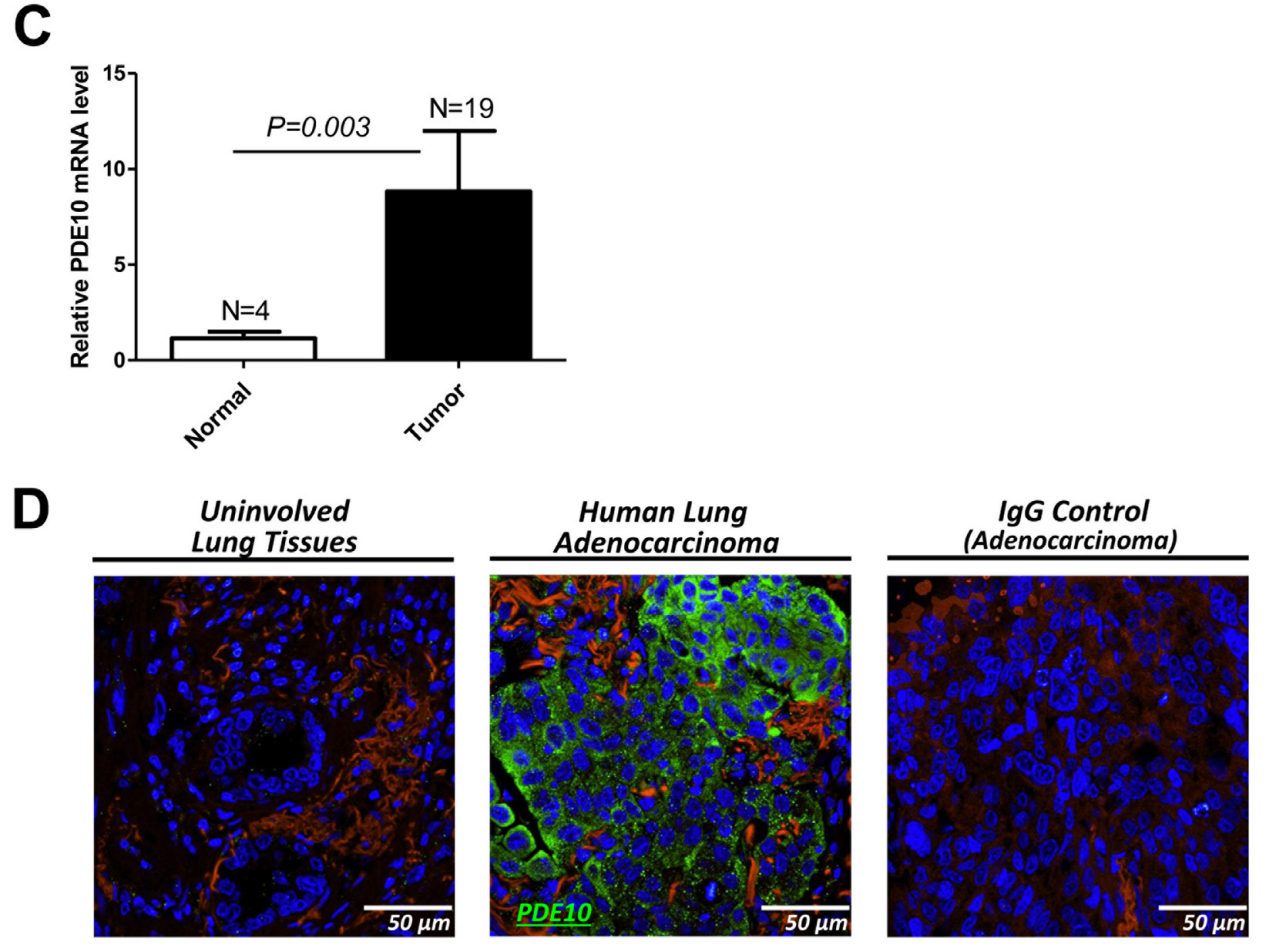
PDE10 overexpression in human lung tumor cells **A.** Protein levels of eight major cGMP-degrading PDE isoforms, 1A, 2A, 3A, 3B, 5A, 9A, 10A and 11A, were measured in five NSCLC cell lines (H460, H1299, HOP62, A549 and H1975) and NHAECs by Western blotting. The frame highlights a protein band at the expected 90 kD molecular weight for PDE10 and increased levels in NSCLCs as compared with NHAECs. GAPDH was used as the loading and blotting control for the comparison of different PDE isoforms among these cell lines. Each isoform of PDE was detected by isoform-specific antibodies and the molecular weight of protein was indicated. **B.** The ratio of mRNA levels of PDE10 and other cGMP PDEs are compared between NHAEC and NSCLC (HOP62) cells by quantitative real time RT-PCR (qPCR). The relative amount of PDE mRNAs were normalized by PDE1A for the comparison of different PDE isoforms after measured using TATA-box binding protein (TBP) as the qPCR control between two cell lines. **C.** qPCR of patient samples show increased PDE10 mRNA levels in lung cancers (*n* = 19) vs. normal lung tissue (*n* = 4). Data are represented as mean ± SEM, *P* = 0.003 by F test. **D.** Specimens of primary human lung adenocarcinoma (*middle*) shows positive staining of PDE10 (green, Alexa 488) but negative staining in uninvolved normal lung tissues (*left*) with the autofluorescence (orange) and nuclear staining (DAPI).

The expression level of PDE10 and other cGMP degrading PDE isozymes was determined in a panel of human NSCLC cell lines, including H460, H1299, HOP62, A549 and H1975, and compared with primary cultures of normal human airway epithelial cells (NHAECs). Western blotting using PDE isozyme specific antibodies demonstrated that PDE10 was abundantly expressed in all five NSCLC cell lines, but undetectable in NHAECs (Figure 1A). Other cGMP degrading PDE isozymes, including PDE1A, 2A, 3A, 3B, 5A, 9A, and 11A were either not expressed in all of the NSCLC cell lines or did not show an appreciable difference in protein expression levels compared with NHAECs. To determine if higher PDE10 protein levels are attributed to increased transcriptional activity, levels of mRNA for the cGMP PDE isozymes expressed in HOP62 cells were measured by qRT-PCR using isozyme-specific primers and compared with mRNA levels of these same isozymes in NHAECs. As shown in Figure 1B, a large difference in PDE10 mRNA levels was detected between HOP62 lung tumor cells and NHAECs, while mRNA levels of other PDE isozymes, including PDE1, 2, 5, and 9 showed relatively minor differences.

PDE10 mRNA levels were also measured in cDNA arrays obtained from human lung tumor specimens by qRT-PCR and compared with levels in normal lung tissues. Consistent with results from cell lines, PDE10 mRNA levels were elevated in lung tumors (*n* = 19) compared with normal lung tissues (*n* = 4) as shown in Figure 1C. Increased PDE10 levels were confirmed by immunofluorescence microscopy in which labeling was readily apparent in human lung adenocarcinomas as shown in Figure 1D, while uninvolved lung tissue showed appreciably less labeling. Consistent with previous studies of the subcellular distribution of PDE10 in colon tumor cells [32], PDE10 was enriched primarily in the cytoplasm, although membrane labeling was also apparent.

### PDE10 knockdown suppresses lung tumor cell growth and colony formation

**Figure 2:**
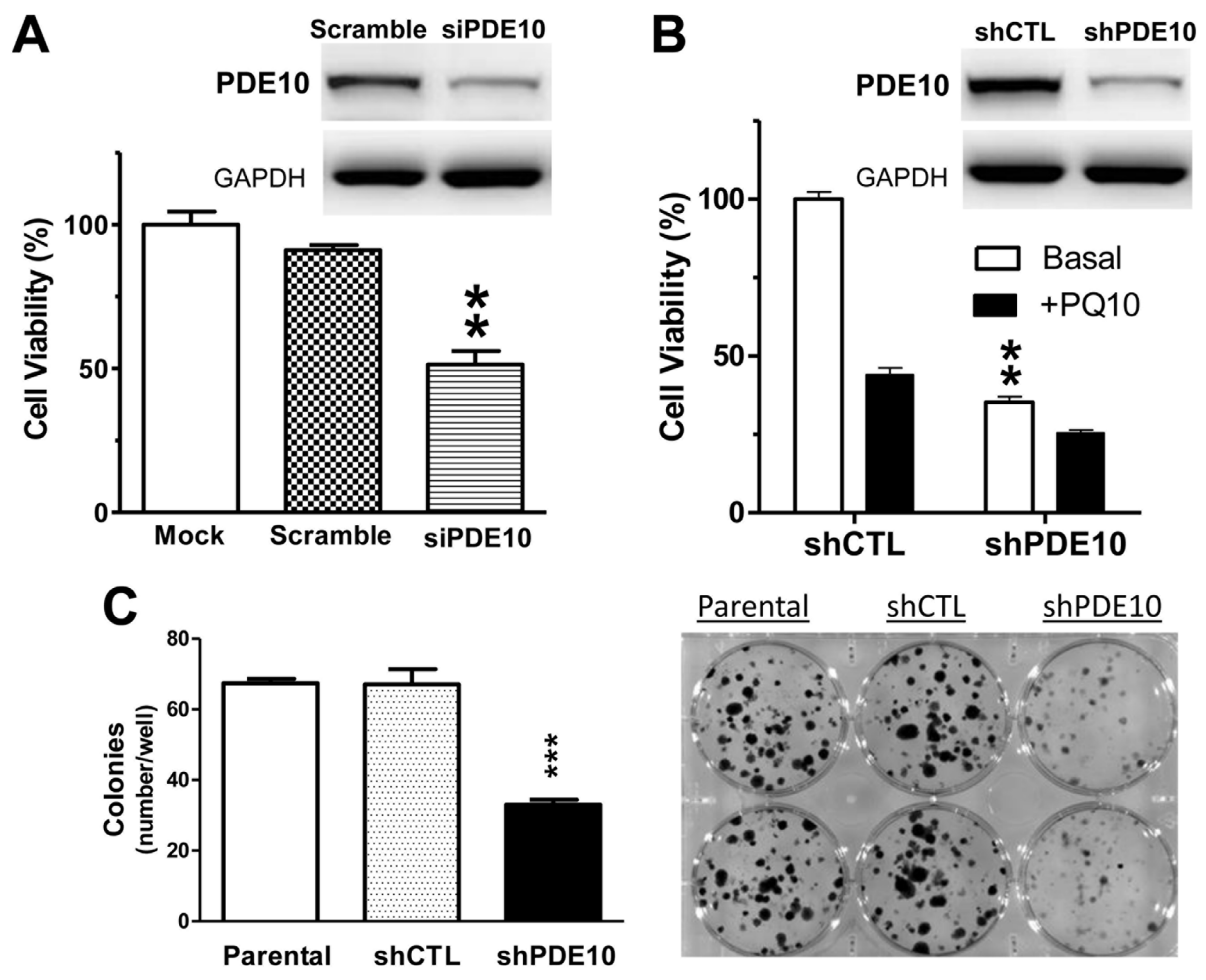

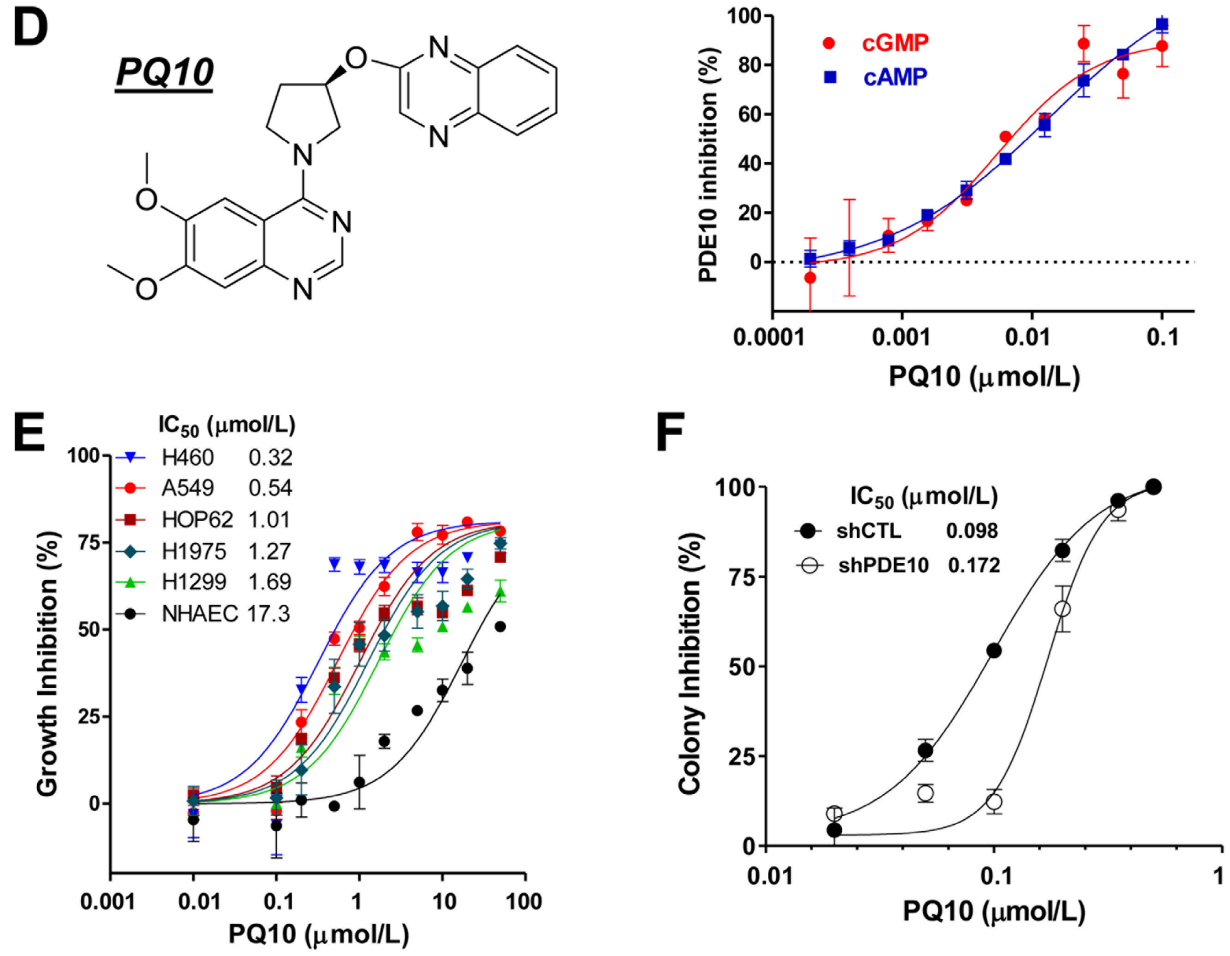

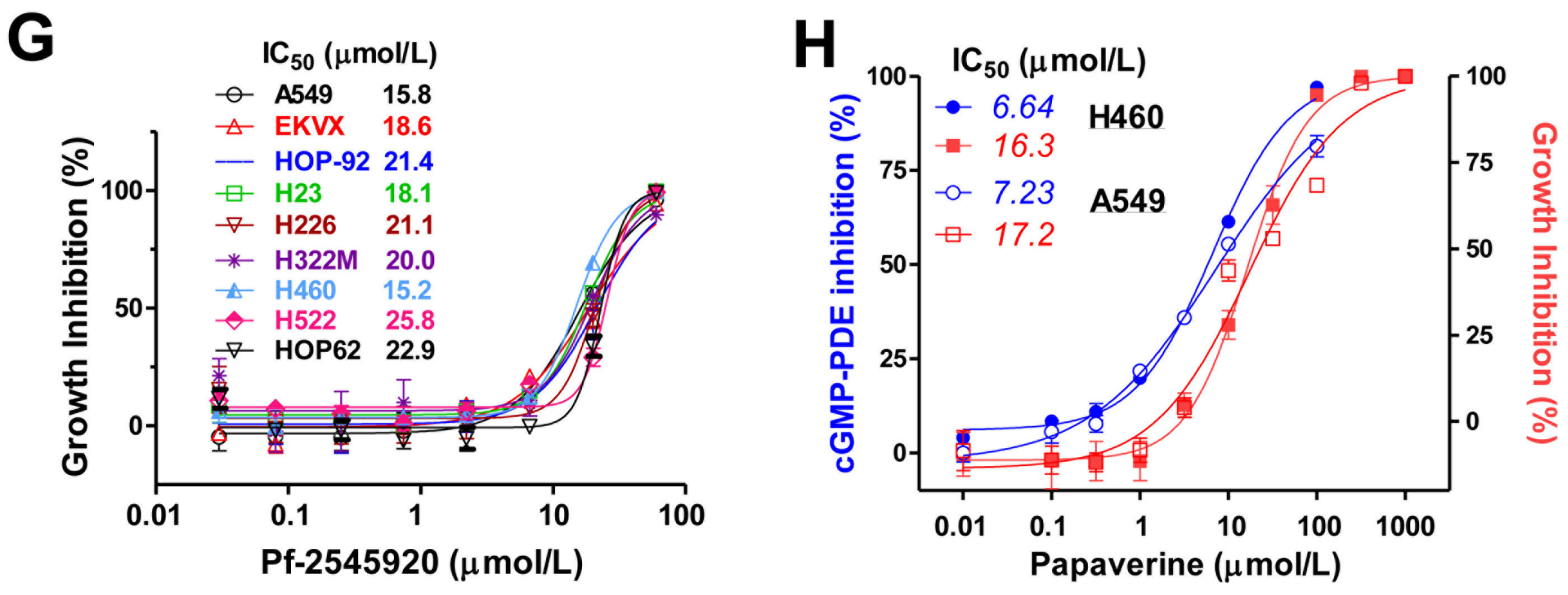
PDE10 inhibition suppresses lung tumor cell growth and colony formation **A.** Genetic silencing PDE10 expression by transient knockdown with PDE10-specific siRNA (siPDE10) selectively inhibited NSCLC HOP62 cell growth as compared with parental cells (mock transfection) or HOP62 cells transfected with control siRNA (scramble). **B.** Stable knockdown of PDE10 by shRNA (shPDE10) in HOP62 cells also inhibited growth and attenuated the response to the PDE10 selective inhibitor, PQ10 (2 µmol/L), as compared with shRNA vector controls (shCTL). *Insets of A/B*, Western blotting confirmed that PDE10 protein levels in HOP62 cells were reduced after siRNA or shRNA treatment as compared with scrambled or shCTL control. **C.** Knockdown PDE10 by shRNA inhibited colony formation in HOP62 NSCLC cells. Stable PDE10 knockdown and parental/shCTL controls of HOP62 cells were grown in culture medium for 14 days followed by staining with crystal violet. Data analysis of colony numbers is shown in the left panel, while representative images are shown in the right panels. **D.** Chemical structure of PQ10 and inhibition of PDE10 by PQ10. **E.** Concentration-dependent growth inhibitory activities and IC_50_ values of PQ10 in NSCLCs vs. NHAECs. **F.** PQ10 inhibited colony formation in HOP62 cells, while PDE10 knockdown cells displayed reduced sensitivity. Results of A∼F represent one of three repeated experiments. Data are represented as mean ± SEM, ** *P*<0.01, *** *P*<0.001 vs. parental, scramble siRNA or shCTL controls. **G.** NSCLC lung cancer cell panels showed growth inhibition and IC_50_ values after a treatment with another PDE10 selective inhibitor, Pf-2545920. **H.** Papaverine inhibited cell growth and cGMP-PDE activities in cell lysates from H460 and A549 NSCLC cells.

To study a potential functional role of PDE10 in lung tumor cell proliferation or survival, PDE10 protein levels were suppressed by transient transfection of HOP62 lung tumor cells with PDE10-specific siRNA. A reduction of PDE10 protein levels as determined by Western blotting coincided with an approximate 50% reduction of viable cell number following three days of transfection in comparison to parental HOP62 cells or HOP62 cells transfected with scrambled siRNA (Figure 2A and inset). Stable knockdown of PDE10 using a specific shRNA resulted in a 70% reduction of viable cell number relative to control cells (Figure 2B). Western blotting showed a greater reduction of PDE10 protein levels in the stable knockdown cells by shRNA (inset, Figure 2B) as compared with transient transfection by siRNA. Stable knockdown by PDE10 shRNA also significantly reduced colony formation of HOP62 cells in which a greater than 60% reduction of colony numbers was observed in PDE10 knockdown HOP62 cells compared with parental and shRNA vector control HOP62 cells (Figure 2C).

### PDE10 inhibitors suppress lung tumor cell growth and colony formation

To further study the role of PDE10 in lung cancer cells, we synthesized a previously reported PDE10 inhibitor referred to as PQ10 [29, 34] with a structure as shown in Figure 2D and evaluated its ability to inhibit lung tumor cell growth. Initial studies were performed to determine the PDE10 isozyme selectivity of PQ10 using recombinant PDE isozymes. PQ10 inhibited PDE10 with IC_50_ values for cGMP and cAMP of 0.005 and 0.013 µmol/L, respectively, as shown in Figure 2D. Appreciably higher concentrations in the micromolar range were required to inhibit other PDE isozymes, demonstrating a high degree of selectivity for PDE10 (Table 1). PQ10 inhibited the growth of human lung tumor cells with IC_50_ values ranging from 0.32 -1.69 µmol/L, resulting in a maximum reduction of viable cell number greater than 75% following 3 days of treatment (Figure 2E). By comparison, NHAECs having reduced levels of PDE10 were as much as 50 fold less sensitive to PQ10 treatment, resulting in an IC_50_ value of 17.3 µmol/L in growth assays conducted under identical conditions as lung tumor cell lines (Figure 2E). PQ10 also caused a concentration-dependent inhibition of colony formation in HOP62 cells with an IC_50_ value of 0.098 µmol/L following 14 days of treatment (Figure 2F).

**Table 1:** PDE isozyme selectivity of PQ10

PDE Family	Substrate	IC_50_ of PQ10	
		cGMP (µmol/L)	cAMP (µmol/L)
1A	cGMP/cAMP	8.8	29.2
2A	cGMP/cAMP	3.7	7.0
3A	cGMP/cAMP	0.32	0.47
4B	cAMP	ND	17.8
5A	cGMP	1.8	ND
6A/B	cGMP	Inactive	ND
7A	cAMP	ND	Inactive
8A	cAMP	ND	Inactive
9A	cGMP	Inactive	ND
10A	cGMP/cAMP	0.005	0.013
11A	cGMP/cAMP	13.2	30.1

ND: not determined; Inactive: >100 µmol/L shows no inhibition.

To confirm that PDE10 inhibition is responsible for the tumor cell growth inhibitory activity of PQ10, HOP62 cells stably transfected with PDE10-shRNA were used to measure their sensitivity to PQ10 and compared with HOP62 cells transfected with vector alone. As shown in Figure 2B, PQ10 effectively inhibited the growth of vector control cells, while PDE10 knockdown cells were appreciably less sensitive to treatment. Similar differences were found in PQ10 treated HOP62 cells with PDE10-shRNA in colony formation assays (Figure 2F). For example, PQ10 treatment on HOP62 vector control cells at a concentration of 0.1 µmol/L reduced colony formation by 54.4% compared with control cells, while treatment of PDE10 knockdown cells with PQ10 resulted in only a 12.3% reduction of colony formation relative to control cells.

Similar growth inhibitory activity was measured with two other known PDE10 inhibitors, Pf2545920 and papaverine, as shown in Figure 2G and Figure 2H, respectively. Pf2545920 effectively inhibited the growth of a panel of nine human lung tumor cell lines derived from the NCI-60 panel. Papaverine also inhibited growth of NSCLC cells with IC_50_ values comparable with IC_50_ values for inhibition of total cGMP PDE enzymatic activity in whole cell lysates.

### PQ10 increases intracellular cGMP levels at concentrations that inhibit tumor cell growth

**Figure 3:**
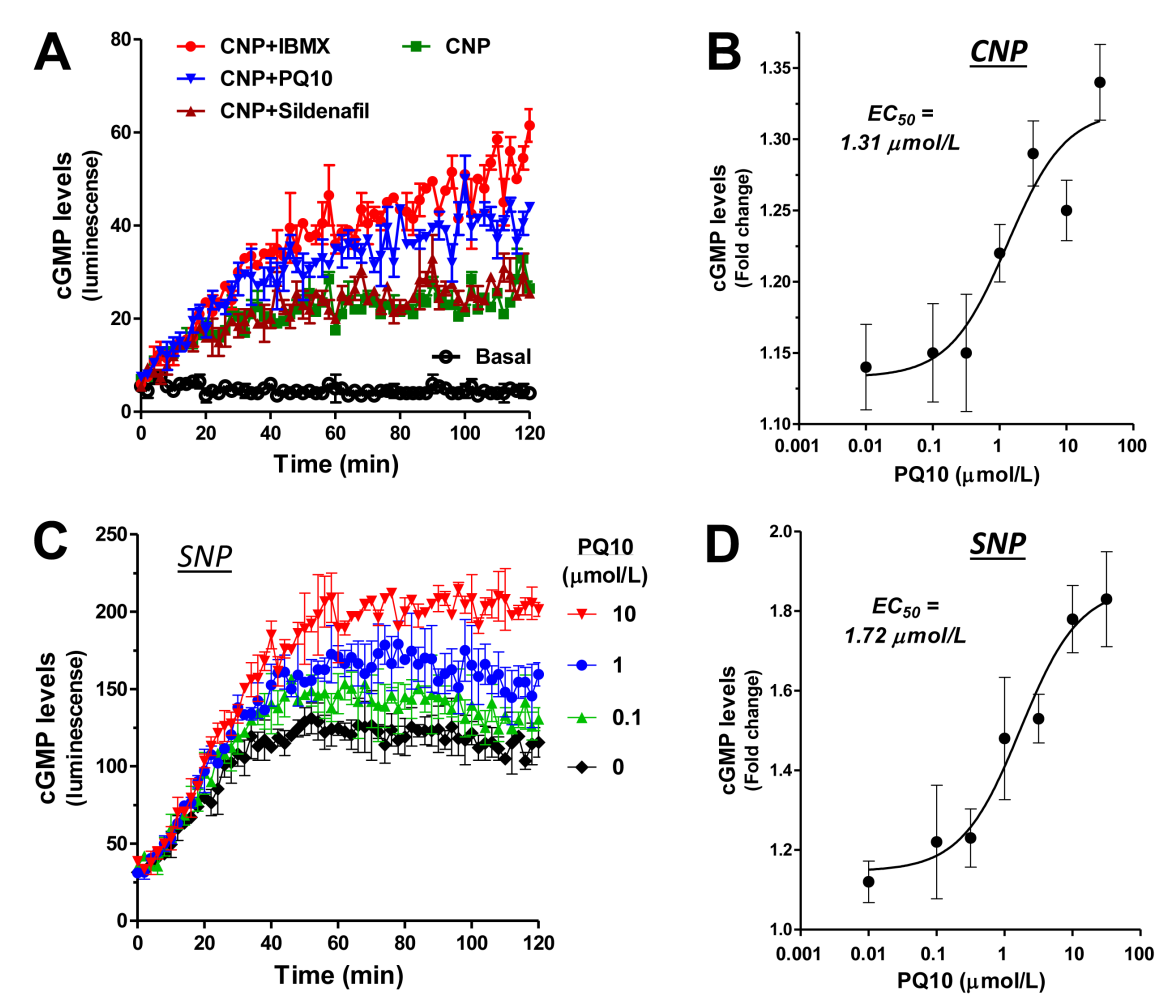

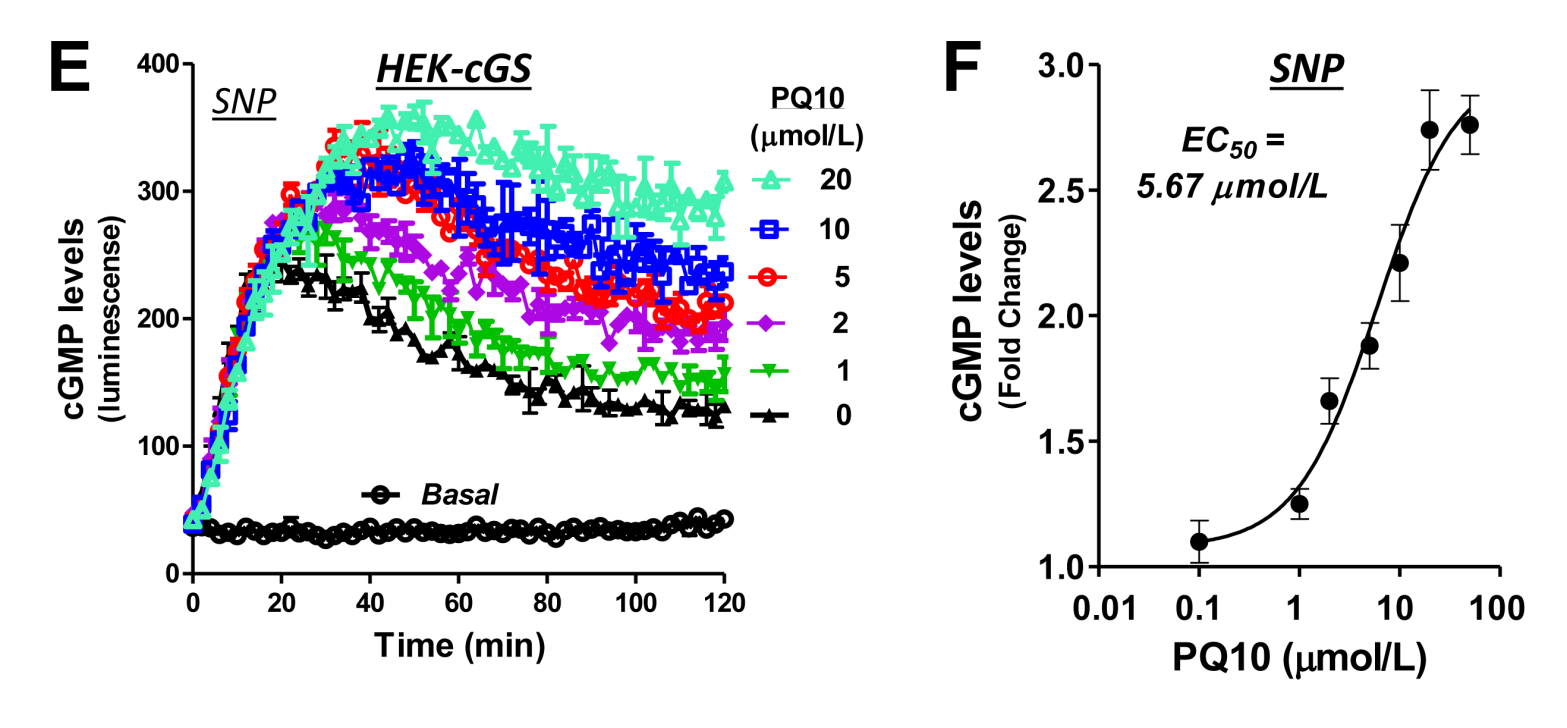
PDE10 inhibition elevates intracellular cGMP levels in NSCLCs as determined by a biosensor assay **A.** Measurement of intracellular cGMP levels in live H1299 NSCLC cells with a stable transfection of cGMP biosensor (cGS) under a stimulation of particulate GC activator, CNP (100 nmol/L), in combined with PQ10 (10 µmol/L) and sildenafil (1 µmol/L, PDE5 inhibitor). PDE isozymes non-selective inhibitor IBMX (100 µmol/L) was used as positive control. **B.** EC_50_ calculation of PQ10 after CNP stimulation. **C.** and **D.** A similar dose-dependent manner and EC_50_ value of PQ10 was present in H1299 biosensor cells under a stimulation of soluble GC activator, sodium nitroprusside (SNP, 100 µmol/L). **E.** and **F.** PQ10 induced a similar manner of increases in intracellular cGMP levels in HEK293 biosensor cells under SNP (100 µmol/L) but the EC_50_ value was about 3.3-fold higher than H1299 cells transfected with the cGMP biosensor. Cells were cultured in 96 well plates to 80-90% confluence and were used for the biosensor assay which involved continuous measurements of luminescence values every two minutes for 2 hours. All cells used for these studies were cultured under similar conditions to obtain IC_50_ and EC_50_ values of PDE activities measurements in cell lysates, growth inhibition assay and biosensor analysis.

To study the effects of PQ10 on cGMP signaling, intracellular cGMP levels were measured in lung tumor cells using a luciferase-based biosensor assay that allowed for real-time measurements of cGMP levels in live cells following treatment [35]. The luciferase construct was transfected into human H1299 lung tumor cells treated with PQ10. As shown in Figure 3A-3D, PQ10 caused a rapid increase in cGMP levels within 1 hour of treatment that was sustained for the duration of the experiment at 2 hours. The EC_50_ values of PQ10 to induce cGMP elevation were 1.31 µmol/L and 1.72 µmol/L for CNP and SNP (Figure 3B and Figure 3D), respectively, which coincided with the IC_50_ value of 1.69 µmol/L for PQ10 to inhibit the growth of H1299 lung tumor cells (refer to Figure 2E). PQ10 also increased cGMP levels in HEK293 cells transfected with the cGMP biosensor in combination with SNP (Figure 3E and Figure 3F).

The PDE5 specific inhibitor, sildenafil, was tested under the same conditions in the biosensor assay and provided a useful negative control having no effect on the growth of H1299 cells (not shown) that do not express PDE5 (refer to Figure 1A). Sildenafil at a concentration of 300-fold higher than its IC_50_ for PDE5 inhibition did not affect luminescence values as determined by the biosensor assay (Figure 3A). In addition, the non-selective PDE inhibitor, IBMX, increased luminescence levels in H1299 cells (Figure 3A), which provided additional evidence that the activity of PQ10 as measured in the biosensor assay was associated with a rise in intracellular cGMP levels.

### PQ10 activates PKG at concentrations that inhibit tumor cell growth

**Figure 4:**
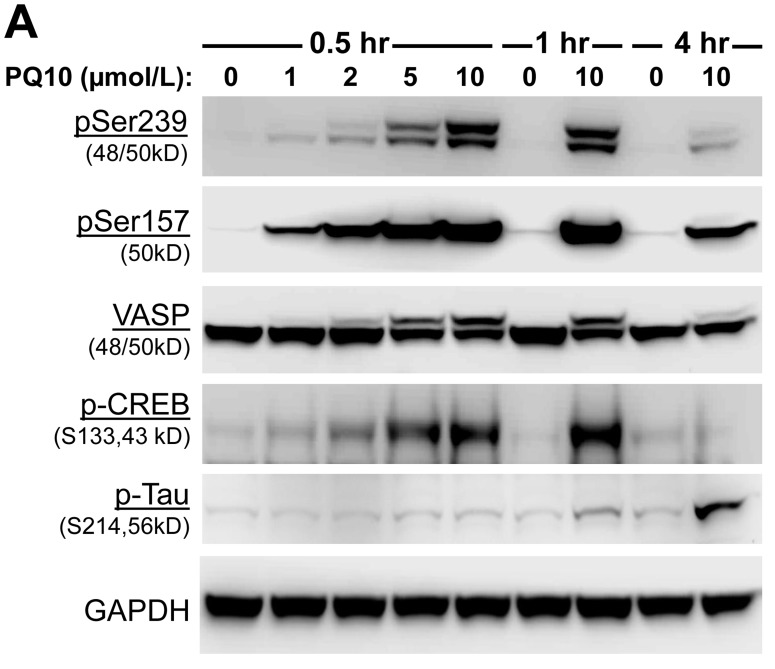

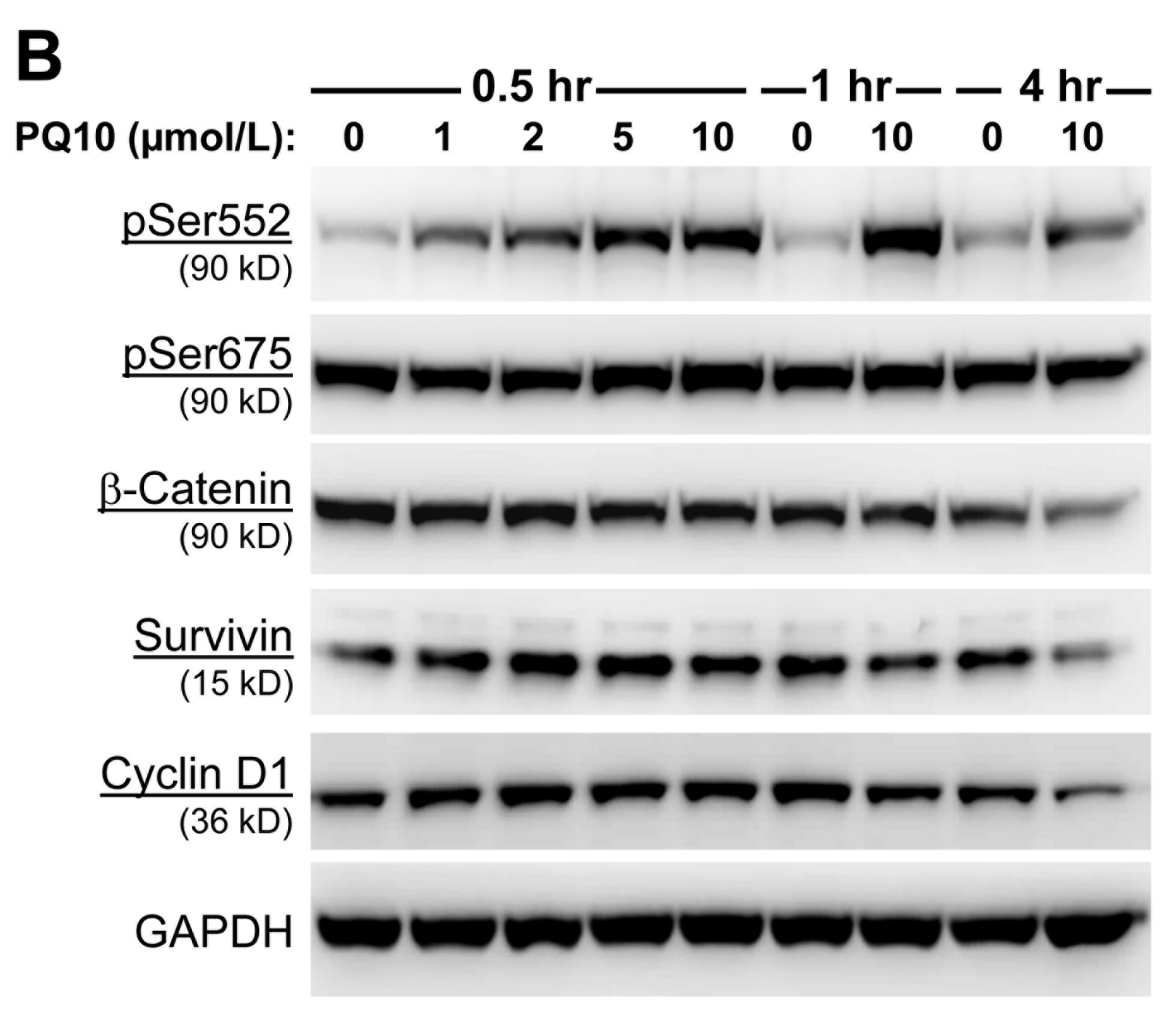

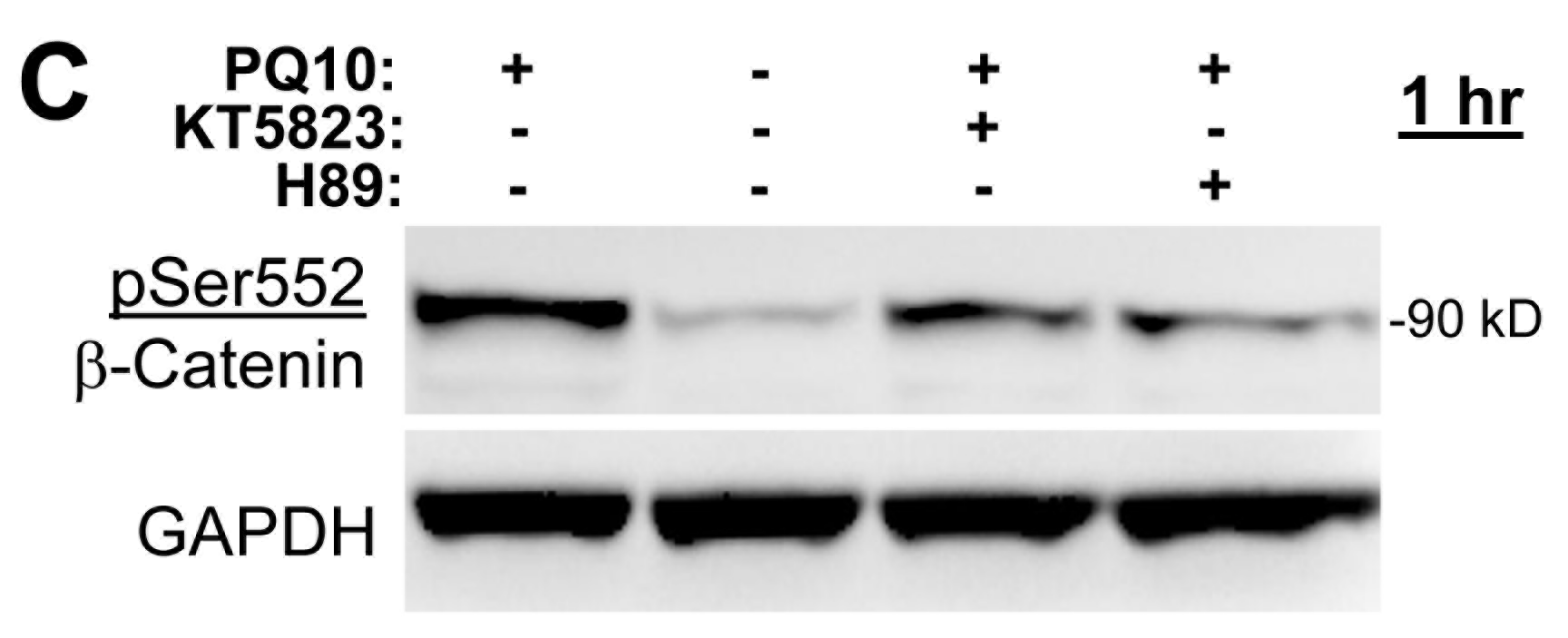

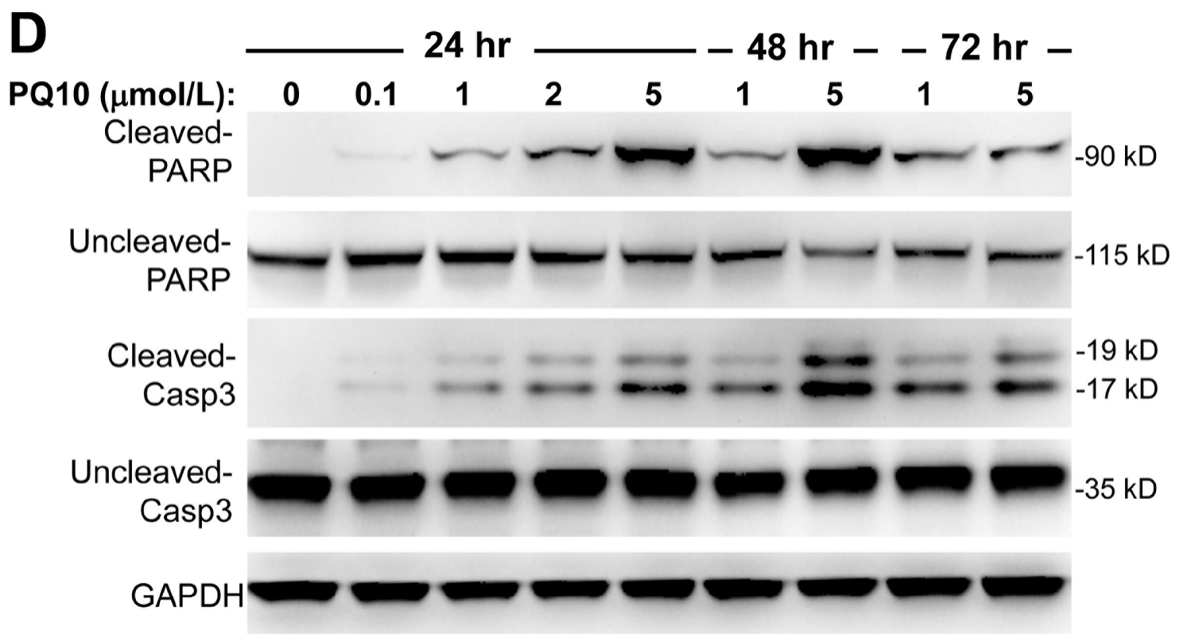

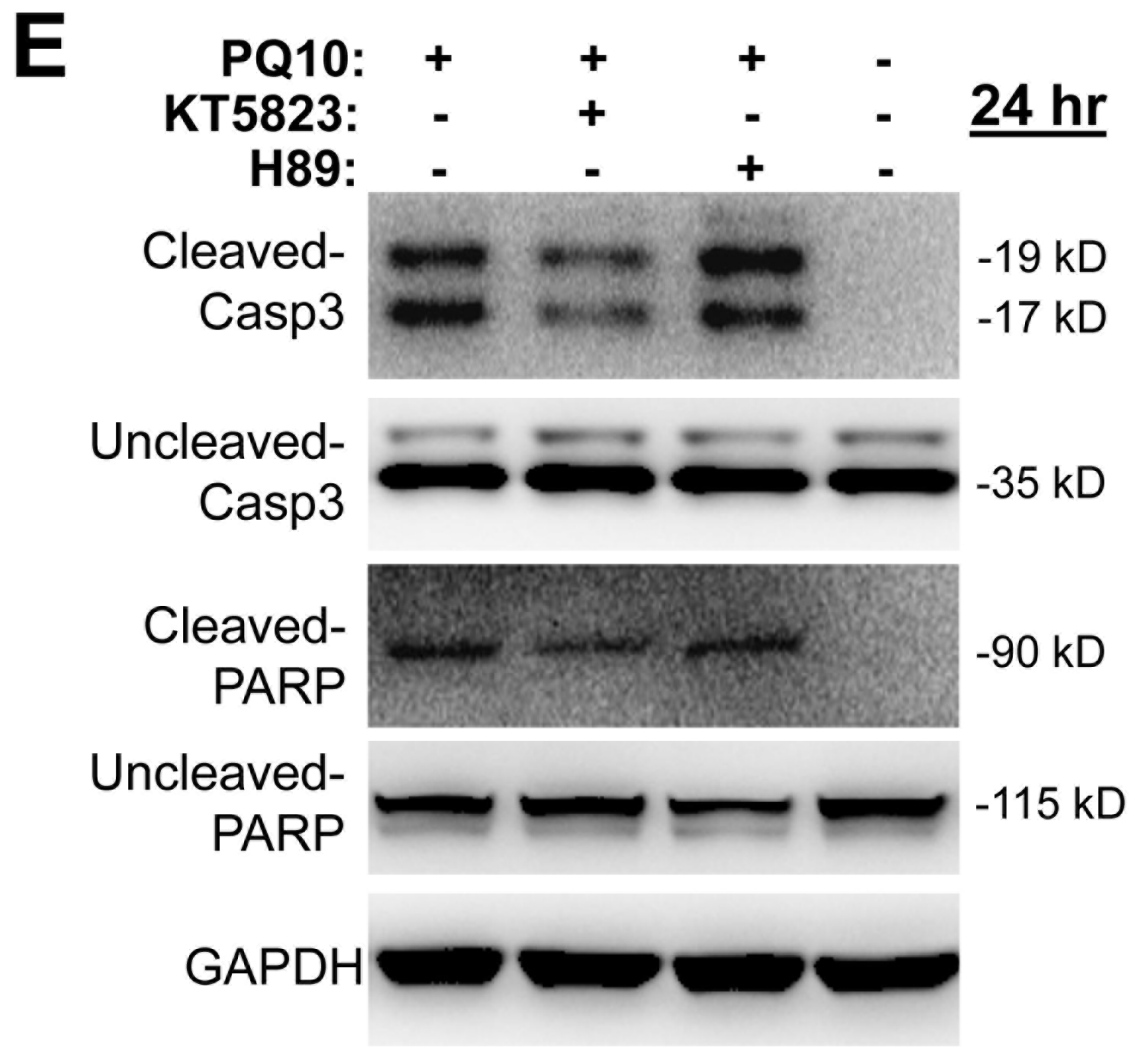
PQ10 activates PKG/PKA signaling, reduces β-catenin expression and induces apoptosis in NSCLC **A.** Site-specific phosphorylation of vasodilator-stimulated phosphoprotein, VASP (Ser157 and Ser259), cAMP response element-binding protein, CREB (Ser133), and microtubule-associated protein, Tau (Ser214) in H1299 cells were measured for concentration-dependent and time-related phosphorylation manners after PQ10 treatments. **B.** PQ10 initiated a phosphorylation of β-catenin at Ser552 but did not show a change at Ser675 after 0.5∼1 hour treatments. PQ10 also reduced total β-catenin, survivin and cyclin D1 expression after 4 hours of treatment. **C.** At 1 hour of treatment, PKG and PKA inhibitor, KT5823 (0.1 µmol/L) and H89 (1 µmol/L) showed partial inhibition of PQ10 (2 µmol/L) induced Ser552 phosphorylation of β-catenin. **D.** PQ10 induced apoptosis in H1299 NSCLC cells. The cleaved nuclear poly ADP ribose polymerase (PARP) and caspase 3 (Casp3) proteins were detected by Western blotting after 24∼72 hours of treatment with different doses of PQ10. **E.** At 24 hours of treatment, PKG-inhibitor, KT5823, showed inhibition of PQ10 induced-cleaved Casp3 and PARP, but PKA-inhibitor H89 was ineffective.

As previously reported, PKG activation occurs in response to an elevation of intracellular cGMP levels and appears to be the major signaling pathway responsible for the tumor cell growth inhibitory activity of cGMP PDE inhibitors [9, 14, 32, 33]. Experiments were therefore conducted to determine if PQ10 can activate PKG by Western blotting using a phospho-specific antibody against phosphorylated serine239 of vasodilator-stimulated phosphoprotein (pS239-VASP), a known cGMP-activated PKG specific site of phosphorylation and known biomarker of PKG activation [36]. As shown in Figure 4A, PQ10 treatment of H1299 lung tumor cells caused a rapid and concentration-dependent increase in pSer239-VASP levels without affecting total VASP protein levels. The effect occurred within 30 minutes of treatment, which paralleled increased levels of intracellular cGMP as measured by the biosensor assay (refer to Figure 3A-D). The effective concentration range was also similar to EC_50_ and IC_50_ values required for increasing cGMP levels and inhibiting H1299 tumor cell growth, respectively. Given that PDE10 can also hydrolyze cAMP, we also determined if PQ10 can simultaneously activate PKA in H1299 cells using an antibody against pSer157-VASP, a known cAMP-activated PKA specific site of VASP phosphorylation. In addition, we used antibodies directed against phosphorylation sites on cAMP response element-binding protein (pSer133-CREB) and the microtubule-associated protein, Tau (pSer214-Tau) that are known to be phosphorylated by either PKA or PKG [37-39]. Consistent with the capacity of PDE10 to hydrolyze cAMP and cGMP, Western blotting showed that PQ10 increased levels of pSer157-VASP and pSer133-CREB in H1299 cells within 30 minutes of treatment, but longer treatment times of 1∼4 hours were required to increase pSer214-Tau levels. These experiments show that PQ10 can activate both PKG and PKA signaling in lung tumor cells and are consistent with effects that are expected from a PDE10 inhibitor.

### PQ10 induces the phosphorylation and degradation of β-catenin

Previous studies have reported that PKG can phosphorylate β-catenin to suppress T-cell factor (TCF) transcriptional activity by inducing ubiquitin-mediated degradation [9, 31-33, 40, 41]. To determine if PQ10 can suppress oncogenic β-catenin signaling, we measured the phosphorylation of β-catenin in H1299 lung tumor cells in response to PQ10 treatment. As shown in Figure 4B, PQ10 increased the phosphorylation of β-catenin at pSer552 in a concentration-dependent manner at levels that were effective for activating PKG. However, there was no change in the phosphorylation level of β-catenin at pSer675, which is a known site of PKA-conserved phosphorylation [42]. Other experiments in which H1299 cells were pretreated with the PKG inhibitor, KT5823, or the PKA inhibitor, H89, showed partial inhibition of PQ10-induced phosphorylation β-catenin at pSer552 (Figure 4C), although the effective concentration of KT5823 was 10-fold lower than H89. Along with the inability of PQ10 to increase the phosphorylation of β-catenin at pSer675, these results suggest that PKG activation may be the predominant signaling pathway to phosphorylate β-catenin in response to PQ10 treatment and to inhibit tumor cell growth, which is supported by previous reports that cAMP elevation and PKA activation do not appear to be essential for the growth inhibitory activity of PDE10 inhibitors [31-33].

To determine if PDE10 inhibition can induce β-catenin degradation as previously reported for other cGMP PDE inhibitors [9, 13, 14, 33, 35], H1299 lung tumor cells were treated with PQ10 and total levels of β-catenin were measured by Western blotting as shown in Figure 4B. While β-catenin was rapidly phosphorylated within 30 minutes of treatment, total β-catenin levels began to decrease after 4 hours of treatment. Coinciding with the suppression of β-catenin levels, PQ10 treatment reduced the levels of cyclin D1 and survivin (Figure 4B), which are known regulated by β-catenin/Tcf transcriptional activity [31-33].

To determine if the suppression of cyclin D1 and survivin levels by PQ10 can induce apoptosis, cleaved PARP and caspase 3 levels were measured in H1299 cells treated with PQ10. As shown in Figure 4D, PQ10 treatment of H1299 cells increased the levels of cleaved PARP and caspase 3 from concentrations as low as 0.1 µmol/L following 24 hours of treatment. The effect of PQ10 reached a maximum at a 5 µmol/L concentration following 48 hours of treatment. Additional experiments showed that PKG inhibitor, KT5823, but not the PKA inhibitor, H89, attenuated the activation of PARP and caspase 3 by PQ10 following 24 hours of treatment (Figure 4E). These results suggest that PKG activation and PKG-mediated phosphorylation of β-catenin at pSer552 may mediate the growth inhibitory activity of PQ10, while PKA activation may not play a significant role.

### PQ10 suppresses RAS-activated RAF/MAPK signaling

**Figure 5:**
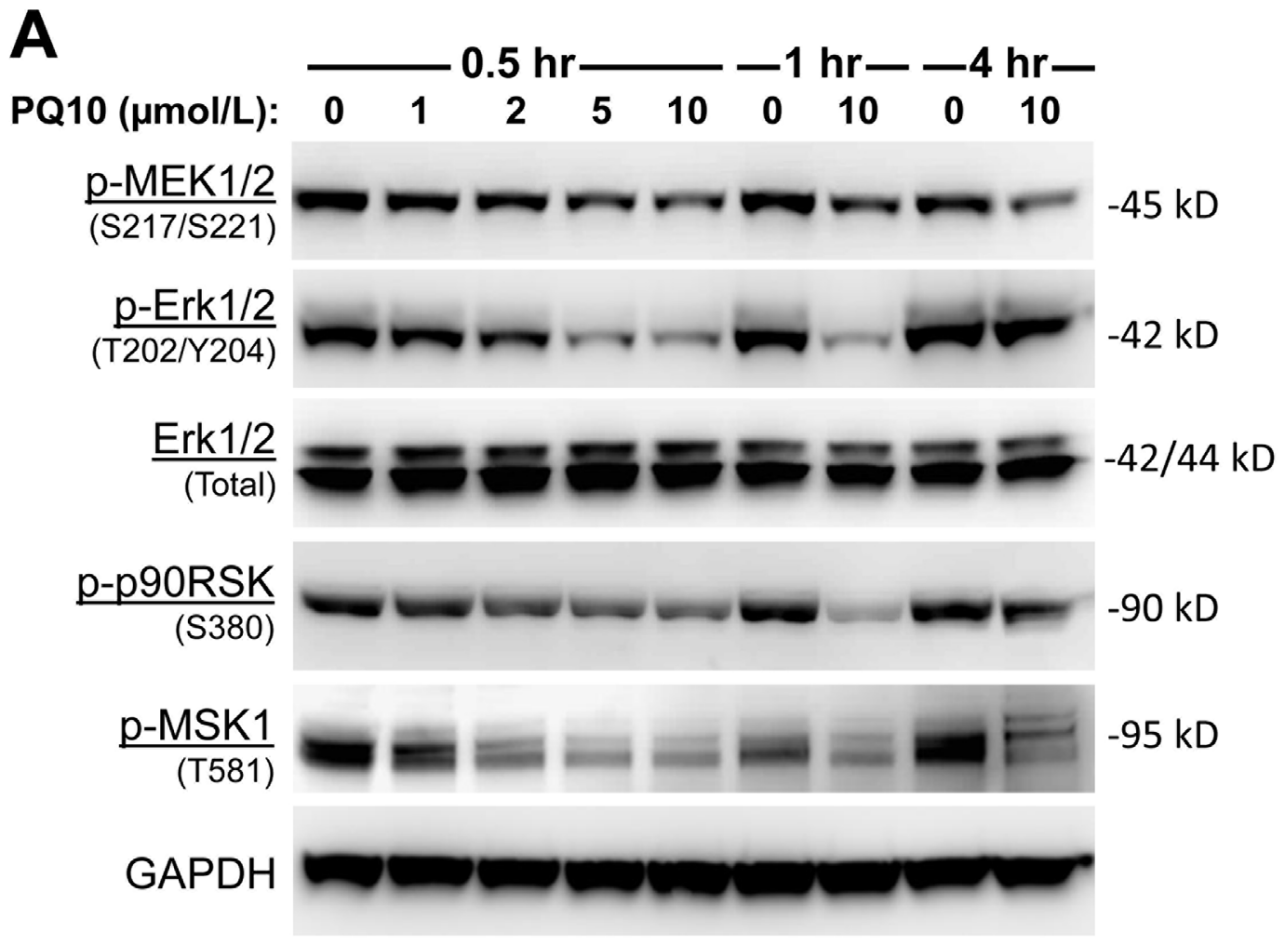

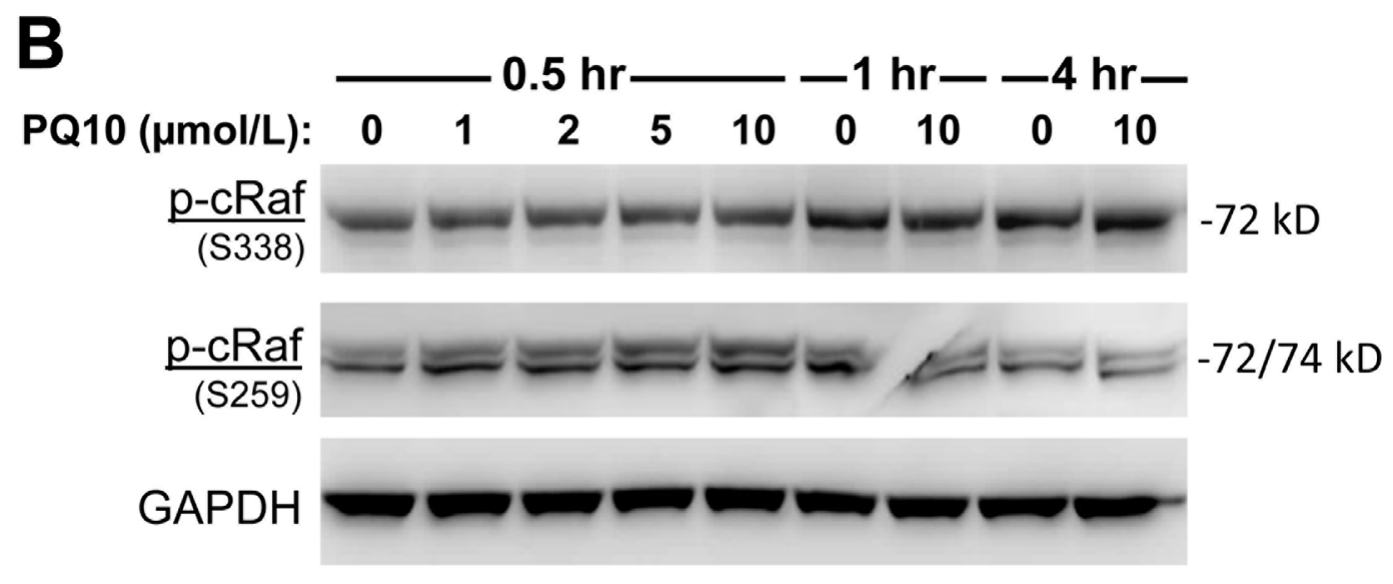
PQ10 suppresses RAF/MAPK signaling **A.** PQ10 showed a dose-dependent and time-related inhibition of ERK1/2 (p42/44) phosphorylation at T202/Y204 in H1299 NSCLC cells without changing total protein levels. The level of downstream targets of activated nuclear phospho-p90RSK (S380) and phospho-MSK-1 (T581) were significantly reduced after PQ10 treatment that followed the kinetics of pERK1/2 reduction. PQ10 also reduced the phosphorylation level of MEK1/2 (S217/S221), the upstream kinases of ERK1/2. **B.** Phosphorylated c-RAF (S338 and S259) levels were also detected after the same treatment of PQ10.

A high percentage of lung cancers harbor mutations in K-RAS that activate the RAF/MAPK pathway to stimulate tumor cell proliferation and survival. Previous studies showing that cGMP can suppress MAPK signaling by inhibiting RAF kinase activation [43] led us to determine the effect of PQ10 treatment on MAPK signaling in H1299 lung tumor cells. PQ10 treatment was found to cause a concentration- and time-dependent inhibition of MAPK signaling as evident by reduced levels of ERK1/2 (p42/44) phosphorylation at T202/Y204 (Figure 5A). The effect occurred rapidly within 30 minutes of PQ10 treatment that paralleled the kinetics of PKG activation. The level of phospho-p90RSK (Ser380), an essential downstream target of activated nuclear ERK1/2, was also reduced by PQ10 treatment with similar kinetics. The same effect was observed for phospho-MSK-1 at T581, another major downstream target of activated ERK1/2 [44]. PQ10 also inhibited the phosphorylation of upstream MEK1/2 (pSer217/Ser221). Consistent with a mechanism of growth suppression resulting from PDE10 inhibition that does not involve PKA signaling, PQ10 did not affect the phosphorylation of c-RAF at serine 338 and serine 259 as shown in Figure 5B. Given that these residues are known to be phosphorylated by PKA [45, 46], our results provide further evidence for a role of cGMP/PKG signaling in the growth inhibitory activity resulting from PDE10 inhibition.

## DISCUSSION

We report here that PDE10 mRNA and protein levels are elevated in human lung cancer cell lines and lung tumors compared with primary cultures of normal airway epithelial cells and normal lung tissues, respectively. Genetic knockdown of PDE10 by either siRNA or shRNA strongly suppressed growth and colony formation of NSCLC cell lines. Three known inhibitors of PDE10, PQ10, Pf2545920, and papaverine, suppressed lung tumor cell growth by a mechanism that was confirmed to be mediated by PDE10 inhibition. PDE10 was not detected in normal airway epithelial cells, which displayed reduced sensitivity to PQ10 compared with NSCLC cells. PQ10 caused a rapid and sustained increase in intracellular cGMP levels as measured by a biosensor assay and activated PKG in lung tumor cells as measured by VASP phosphorylation. These effects of PQ10 occurred within the same concentration range as required for growth inhibition. PQ10 increased the phosphorylation of β-catenin with the same kinetics as VASP phosphorylation, while longer treatments reduced protein levels of β-catenin, as well as cyclin D1 and survivin levels, which preceded the activation of PARP and caspase 3 enzymes required for apoptosis. PDE10 inhibition by PQ10 also suppressed RAS-activated RAF/MAPK and downstream pathway components such as p90RSK/MSK-1 phosphorylation. Together, these results indicate that PDE10 expression is elevated in lung cancer cells relative to normal airway epithelial cells and essential for lung tumor cell proliferation and survival.

PDE10 mRNA and protein levels were essentially undetectable in NHAEC and normal lung epithelium, which is consistent with the limited expression of PDE10 as previously reported in normal tissues outside the central nervous system [24-26]. To our knowledge, lung tumors have never been previously studied for PDE10 expression. High PDE10 mRNA levels were measured in human lung tumors relative to normal lung tissues. PDE10 protein levels were confirmed by immunolabeling of lung adenocarcinomas using a PDE10 specific antibody and imaged by immunofluorescence microscopy, which showed cytoplasmic and membrane localization. Our results are consistent with recent observations of PDE10 overexpression in precancerous adenomas from the ApcMin/+ mouse model of colon cancer, as well as human colorectal adenocarcinomas [31]. These observations suggest that PDE10 is expressed during lung tumor development and essential for tumor growth.

Specific overexpression of the PDE10 isozyme in lung tumor cell lines was revealed by simultaneous detection of all known cGMP PDE isozymes (except PDE6) by Western blotting using isoform-specific antibodies. These experiments showed that while some isozymes could be detected in certain lung tumor cell lines, none were uniformly expressed in all NSCLC lines but not expressed in NHAEC, as the case of PDE10. Previous studies have shown that the cGMP specific PDE5 isozyme is expressed in various tumor cell lines, including those derived from lung, colon, and breast tumors [10-12]. We detected PDE5 in two of five NSCLC cell lines examined, but the levels were comparable to NHAEC. The PDE5 selective inhibitor, sildenafil has also been reported to inhibit tumorigenesis in an inflammation-driven model of colon cancer, which suggests a potentially important role of PDE5 in tumorigenesis [47]. However, the abundance of PDE10 in tumor cells may limit the sensitivity of tumor cells to PDE5 inhibitors, whereby combined treatment with PDE5 and PDE10 inhibitor or treatment with a dual PDE5/10 inhibitor may hold greater promise as a therapeutic strategy [33, 48]. In addition, we found that PDE9 was expressed in lung tumor cell lines, which is consistent with observations from other investigators reporting effects of PDE9 inhibitors on breast tumor cell growth [49-51]. However, since PDE9 is also expressed in NHAEC, it may provide a “house-keeping” function in the lung epithelium unrelated to tumorigenesis.

Experiments to determine if PDE10 represents a potential therapeutic target for lung cancer were performed using three known small molecule inhibitors of PDE10, including PQ10, Pf2545920 and papaverine. All three compounds effectively suppressed lung tumor cell growth with PQ10 showing the greatest potency. Because of a significance difference between IC_50_ values for PDE10 and growth inhibition, we performed additional experiments to confirm that PDE10 inhibition is indeed responsible for the growth inhibitory activity of PQ10. First, we profiled the inhibitory activity of PQ10 against a full panel of PDE isozymes, which confirmed that the compound is highly selective for inhibiting PDE10. We then showed that the tumor cell growth inhibitory activity of PQ10 occurred within the same concentration range that increased intracellular cGMP levels.

Relative intracellular cGMP levels were measured using a cGMP biosensor assay, which to our knowledge is the first report showing the utility of a luminescence-based assay for measuring cGMP levels in live cancer cells. PQ10 increased intracellular cGMP levels in H1299 cells with kinetics that paralleled the activation of PKG activation as measured by the phosphorylation of VASP at serine 239. Notably, the concentration ranges of both effects as measured by two distinct assay formats were comparable with IC_50_ values for growth inhibition. As compared with lung tumor H1299 cells, treatment of HEK293 cGMP biosensor cells with PQ10 showed an increase in cGMP levels at a higher EC_50_ value, coincident to a higher IC_50_ value for growth inhibition of cells derived from normal tissues (e.g. NHAECs). It is also noteworthy that with such specific cGMP biosensor analysis for PDE10 in H1299 cells, an undetectable change of intracellular cGMP levels was seen for sildenafil treatment at a concentration 300-fold higher than its IC_50_ to inhibit PDE5, in which the cells showed undetectable levels of PDE5 and no effect of sildenafil on growth.

Treatment of NSCLC cells with PQ10 to activate PKG was found to be associated with increased phosphorylation of β-catenin followed by reduction of protein levels. Under the same conditions, PQ10 reduced cyclin D1 and survivin levels, which occurred within a treatment period that preceded the activation of PARP and caspase 3 leading to apoptosis. These observations are consistent with previous reports that PDE10 inhibition can reduce the expression level, nuclear translocation, and TCF transcriptional activity of β-catenin in colon tumor cells [31-33]. Given that the activation of cGMP/PKG signaling has been reported to suppress oncogenic Wnt/β-catenin signaling in colon and breast cancer cells by inhibiting β-catenin expression at mRNA level and β-catenin/CTNNB1 promoter levels [14, 32], a similar mechanism may exist in lung tumor cells. We also report here for the first time that PDE10 inhibition by PQ10 can stimulate the phosphorylation of β-catenin at serine 552, which is considered to be a dual phosphorylation site for PKG and PKA. Interestingly, PQ10 did not affect the phosphorylation of the PKA-conserved site of β-catenin at serine 675. PQ10-induced phosphorylation of β-catenin at serine 552 was shown to be reduced by a PKG inhibitor at lower concentrations than a PKA inhibitor. Moreover, the time course paralleled the kinetics of cGMP/PKG-mediated VASP phosphorylation at serine 239, but occurred prior to the reduction of β-catenin expression. These results support the possibility of a PKG-mediated mechanism to suppress oncogenic Wnt/β-catenin signaling by ubiquitination and proteosomal degradation as previously reported by others [9, 40, 41]. It is unclear if this mechanism is distinct from the action of cAMP on the PKA-conserved phosphorylation at serine 675 of β-catenin as reported in non-tumor cells [42].

Finally, we determined the effect of PDE10 inhibition on the phosphorylation of MEK1/2 and ERK1/2 because of previous reports showing that cGMP/PKG signaling can inhibit RAS-activated RAF/MAPK-kinases [43, 52, 53]. PQ10 was found to inhibit the phosphorylation of MEK1/2 and ERK1/2 in lung tumor cells within the same concentration range that was effective for suppressing β-catenin transcriptional activity. PQ10 also strongly inhibited the phosphorylation of downstream p90RSK and MSK-1, which are important for receptor tyrosine kinase stimulated nuclear transcription and ribosomal translation [44]. In addition, the timeline of PQ10 suppression of RAF/MAPK signaling followed the time required to increase intracellular cGMP levels and activate PKG, which paralleled the time required for β-catenin phosphorylation (Ser552) and the suppression of β-catenin, survivin and cyclin D1 levels, leading to the induction of apoptosis.

In conclusion, PDE10 is overexpressed in lung tumor cells and appears to be essential for lung tumor cell proliferation and survival. The growth inhibitory activity resulting from PDE10 inhibition in lung tumor cells was associated with the elevation of intracellular cGMP levels and activation of PKG to inhibit two important oncogenic pathways involving β-catenin and MAPK signaling. The ability of PDE10 inhibitors to simultaneously suppress both oncogenic pathways may result in greater antitumor efficacy and reduced potential for resistance compared with other targeted drugs that are selective for one or the other pathway. We conclude that PDE10 represents a novel molecular target for the treatment of lung cancer.

## MATERIALS AND METHODS

### Reagents and drugs

The isozyme specific antibodies for PDE1, 2, 3, 9, 10, and 11 were purchased from GeneTex. Other antibodies, including a PDE5 specific antibody and phospho-specific antibodies for protein phosphorylation and their non-phosphorylated forms were purchased from Cell Signaling Technologies. Small interfering RNA (siRNA) targeting human PDE10 (siPDE10) and scramble control siRNA were purchased from Dharmacon and Qiagen as previously described [31]. Papaverine was purchased from Sigma-Aldrich. Pf-2545920 was purchased from Selleck Chemicals, while PQ10 was synthesized at our institution as described previously [34]. DMSO was used as vehicle for all compounds unless otherwise noted. All other reagents were purchased from Sigma-Aldrich or as indicated.

### Cells and cell culture

Human non-small cell lung cancer (NSCLC) cell lines were purchased from the American Type Culture Collection (ATCC) and grown under standard cell culture conditions in RPMI1640 medium containing 5 % fetal bovine serum at 37°C in a humidified atmosphere with 5% CO_2_. All NSCLC cell lines were expanded upon delivery, and numerous vials of low-passage cells were preserved in liquid nitrogen. Cells were passaged in culture no longer than 2 months. Cell line characterization is conducted by ATCC through short tandem repeat profiling. No additional authentication of NSCLC cell lines was conducted except for experimental reasons (e.g. confirmation of PDE10 expression levels and sensitivity to PDE10 inhibitors, etc.). Normal human airway epithelial cells (NHAECs) were obtained from an adult donor and established as primary cultures (P0) from the University of Alabama at Birmingham. NHAECs were grown in serum-free Bronchial Epithelial Cell Basal medium (Lonza) supplemented with growth factors and other nutrients (SingleQuots) for additional passaging (P1∼P3). All cell passages used for experiments were maintained below passage 4.

### Knockdown of PDE10 expressions by siRNA and shRNA

The transient knockdown of PDE10 in NSCLC cells by PDE10-specific siRNA and scramble siRNA control was performed using RNAiMAX reagent (Invitrogen) as previously described [31]. Effects of siRNA on NSCLC cell growth and PDE10 expression levels were measured after 72 hours of treatment. For a stable knockdown of PDE10, GIPZ lentiviral short hairpin RNA (shRNA) vectors targeting human PDE10, nonsilencing GPIZ lentiviral control vector, and transduction kit were purchased from Open Biosystems [31]. Lentiviral particles of shRNA were produced in HEK293T cells (ATCC) according to the manufacturer’s instructions. HOP62 NSCLC cells were transfected by the lentivirus particles followed by puromycin (5 μg/mL) selection. Stable expression cell lines with shRNA of PDE10 (shPDE10) or lentiviral vector control (shCTL) were obtained by limited dilution for single cell clone and confirmed by Western blotting.

### TissueScan lung cancer cDNA array and real-time PCR

TissueScan lung cancer cDNA arrays were purchased from Origene containing 19 lung tumors (with 8 adenocarcinomas as the major subtype) and 4 normal lung tissues that were used to evaluate PDE10 mRNA overexpression in human lung cancer. High quality total RNAs were extracted from pathologist-verified tissues and used for the synthesis of cDNAs for real-time PCR. Real-time PCR was performed using Bio-Rad Chromo 4 system and quantified using the 2^-ΔΔCt^ method as we previously described [31]. PDE10 levels were normalized using β-actin. Human PDE10 primers and RT2 SYBR Green qPCR Mastermix were purchased from Qiagen. The mRNA levels of PDE10 and other PDE isozymes in cultured HOP62 and NHAECs were evaluated by the same method. Primers used for testing the expression of various PDE isoforms in cultured cell studies were self-designed and synthesized. Forward primers are: GCATACAGGGACAACAAACAAC (PDE1A), CACTTCTGCTACCTGCTCTAC (PDE2A), CAGGAAACGGTGGGACATTTA (PDE5A), CACTTGGCTGTCCTAGAGAAAC (PDE9A), GGACAGAGACAAGAAGGATGAA (PDE10A); reverse primers are: GTGGTGATTCTCAA-GGACAGAG (PDE1A), GTCATGACACATGCAGGAAATAA (PDE2A), GAGCTGAGCAT-TATGAAGAACAATAC (PDE5A), GCTCCTCCCTCATCTTCTTAATG (PDE9A), GGATC-TGGGTAAGGGTTGTATAG (PDE10A). The primers for control TATA-binding protein (TBP) are: CTTCGGAGAGTTCTGGGATTG (F) and CACGAAGTGCAATGGTCTTTAG (R).

### Growth assays

Cells were plated in half area 96-well microtiter plates (Corning) at a density of 3, 000 cells per well. For drug treatment, cells were treated with compound or DMSO as vehicle (final concentration at 0.2%). For siRNA assays, cells were transfected with siRNAs under the same condition as described above. The effect of treatment on cell growth was measured using the Cell Titer Glo (CTG) assay (Promega) as specified by the measurement of viable cells based on ATP content after 72 hours standard culture [31]. All growth assay involving NSCLCs and NHAECs were performed in the presence of 5% serum.

### Colony formation assays

For colony formation assays, 200 viable cells were plated in 6-well tissue culture plates and were cultured in RPMI medium supplemented with 5% serum. DMSO (0.2%) as the vehicle and different concentrations of PQ10 were added to the cell culture medium for 14 days of treatment. Colonies were counted after staining with crystal violet as previously described [31].

### cGMP biosensor assay

Stable cell lines with an expression of luciferase-based cGMP biosensor were established from H1299 NSCLC cells and HEK293 cells using a genetically encoded GAF-Luc construct (Promega), containing a cGMP-specific binding domain from human PDE5A GAF-A domain fused to modified firefly luciferase (35). Cells were pre-incubated at room temperature in CO_2_ independent media supplemented with 10% FBS and 5 mmol/L luciferin. For the measurement of intracellular cGMP levels, sodium nitroprusside (SNP) or C-type natriuretic peptide (CNP) in combination with PDE inhibitors were added to initiate the experiment and the increased luminescence values in live cells were detected constantly over a period time of 2 hours.

### PDE assay

The enzymatic activity of recombinant PDE or PDE activities in cell lysates were measured using the IMAP fluorescence polarization (FP) PDE assay (Molecular Devices) as previously described [12, 13]. PDE10 inhibitors and DMSO as the vehicle were added to assay buffer 10 minutes before the incubation with the enzyme reaction. Fluorescence polarization values were measured using Synergy H4 Hybrid Reader (BioTek).

### Western blotting

Whole cell extraction was performed according to a previously described method [12, 23, 35] using cell lysing buffer (Cell Signaling) supplemented with 10 mmol/L NaF for protein phosphorylation studies. Cell lysates were separated by 4-12% gradient, 7% or 10% pre-casting PAGE (Novex/Invitrogen) followed by electrophoretic transfer to a nitrocellulose membrane [13, 23]. Western blotting procedures were performed using Chemiluminescence Reagent (Pierce) and visualized using Chemi Doc Imaging System (BioRad).

### Histological immunofluorescence microscopy

Portions of the lung tissue with tumors were immersed in formalin, embedded in paraffin, and 5 μm sections were placed on glass slides for histological immunofluorescence. Slides were deparaffinized in xylene and rehydrated by decreasing concentrations of ethanol in water. All slides were blocked with background sniper (Biocare) for 30 minutes and incubated overnight in a 1:200 dilution of the primary antibody against the carboxyl terminus of human PDE10A (GeneTex) in diluent solution (Fisher). Slides were then rinsed 3 times with 1X tris-buffered saline (TBS) and incubated in a 1:500 dilution of the Alexa Fluor 488 conjugated secondary antibody (Invitrogen) for 1.5 hours. Slides were counterstained with DAPI (NucBlue Fixed Cell ReadyProbe Reagent; Fisher) and fluorescent mounting medium (Vectashield; Vector) was used to apply the coverslips. Spectral images of fluorescence emission were acquired at 10 nm increments, from 425-685 nm, using an A1R spectral confocal microscope. NIS Elements software (Nikon Instruments, Inc., Melville, NY, USA) was used to acquire all confocal images and hyperspectral analysis was used to identify fluorophores of interest in highly autofluorescent lung tissue as previously described [54].

### Experimental design and data analysis

Drug effects on cell growth and IC_50_ values were determined as described previously [31]. All experiments were repeated a minimum of three times to determine the reproducibility of the results. All error bars represent standard error of the mean (SEM). Statistical analysis was performed using Student’s t test or other as indicated. A *P* value of <0.05 was considered statistically significant.

## Abbreviations

cAMP: cyclic adenosine monophosphate
cGMP: cyclic guanosine monophosphate
cGS: cGMP biosensor
pGC: particulate guanylyl cyclase
sGC: soluble guanylyl cyclase
PDE: phosphodiesterase
PKA: cAMP dependent protein kinase
PKG: cGMP dependent protein kinase
shRNA: short hairpin RNA
siRNA: small interfering RNA
VASP: vasodilator-stimulated phosphoprotein
